# Therapeutic use of botulinum toxin in pain treatment

**DOI:** 10.1042/NS20180058

**Published:** 2018-08-31

**Authors:** Raj Kumar

**Affiliations:** Botulinum Research Center, Institute of Advanced Sciences, Dartmouth, MA 02747, U.S.A.

**Keywords:** Axonal transport, Botulinum Toxin, Central Nervous System, Nociception, Neurotransmitters, Sensory Neurons

## Abstract

Botulinum toxin is one of the most potent molecule known to mankind. A neurotoxin, with high affinity for cholinergic synapse, is effectively capable of inhibiting the release of acetylcholine. On the other hand, botulinum toxin is therapeutically used for several musculoskeletal disorders. Although most of the therapeutic effect of botulinum toxin is due to temporary skeletal muscle relaxation (mainly due to inhibition of the acetylcholine release), other effects on the nervous system are also investigated. One of the therapeutically investigated areas of the botulinum neurotoxin (BoNT) is the treatment of pain. At present, it is used for several chronic pain diseases, such as myofascial syndrome, headaches, arthritis, and neuropathic pain. Although the effect of botulinum toxin in pain is mainly due to its effect on cholinergic transmission in the somatic and autonomic nervous systems, research suggests that botulinum toxin can also provide benefits related to effects on cholinergic control of cholinergic nociceptive and antinociceptive systems. Furthermore, evidence suggests that botulinum toxin can also affect central nervous system (CNS). In summary, botulinum toxin holds great potential for pain treatments. It may be also useful for the pain treatments where other methods are ineffective with no side effect(s). Further studies will establish the exact analgesic mechanisms, efficacy, and complication of botulinum toxin in chronic pain disorders, and to some extent acute pain disorders.

## Introduction

Pain is an unpleasant sensory and emotional experience which substantially reduces the quality of life. This subject has attracted attention from scientists as well as philosophers. Charles Darwin described pain as a ‘homeostatic emotion’ which is essential for the survival of species [1]. Philosopher Rene Descartes described the pain as a sensation which is running from skin to brain [2]. Pain is also viewed as protective and beneficial to recuperation. However, in certain conditions, pain becomes a disease itself. In brief, pain can be perceived as a result of complex neuronal processes which are evoked to set up a new balance between excitation and inhibition. Basically, pain is understood as a response of neuronal cells. Recent advances are developing the concept that pain involves immune cells, glial cells, and astrocytes, which form an integrated network with neuronal circuits to modulate pain. Pain research has uncovered several important neuronal mechanisms.

Pain research is a major health problem. Pharmaceutical industries always look for a better drug for pain, partly due to less understanding of its complex mechanism. Pain therapy is not only poorly managed, but they also lack efficacy. A reasonable and effective pain management strategy requires a basic understanding of following things: (i) knowledge of pain inciting stimuli, (ii) involved neural pathways, (iii) response of nervous systems, and (iv) systemic consequences of pain. Having this knowledge, management of pain will be easy and the use of pharmacologic agents and various hypoanalgesic techniques can be optimized for better management of pain and related consequences. Two types of drugs modulate pain: analgesics and anesthetics. Many of the currently available pain therapies are either inadequate or cause uncomfortable to deleterious side effects. The most clinically used drugs are the opioid family, which include morphine and heroin. But these drugs have several significant side effects, including addiction. Scientists are still looking for better medications. Like solution to every problem, mother nature has also provided some alternatives, such as (i) cone snail venom which is 100-times more potent than existing pain medication, (ii) spider venom has a potent compound which blocks voltage-gated sodium channel 1.7 (Na_v_ 1.7), and (iii) poison from the skin of South American Ecuadorean frog, ABT-594, as powerful painkillers with none of the damaging side effects.

One of the naturally existing potent molecules is Botulinum Neurotoxins (BoNTs). Although BoNTs are extremely toxic molecules, these are now increasingly used for the treatment of disorders related to muscle and glandular hyperactivity. Weakening of muscles due to the peripheral action of BoNTs produces the therapeutic effect. Toxin A is approved in the United States for the treatment of cervical dystonia, blepharospasm, and glabellar lines. Although it is too early to establish observations suggesting analgesic property of botulinum toxin, the toxin is known to modify the sensory feedback loop to the central nervous system (CNS). Further, peripheral injection of BoNT A induces nociceptive behavior in animal models of inflammatory and traumatic neuropathic pain [3–7]. Some observations suggest Botulinum Toxin A is effective in neuropathic pain. Neurogenic pain pathways may have a role in neuropathic pain [8–12]. The process relating to various forms of pain is quite complex, but the basic signaling of painful sensations does not require exocytotic signaling in the peripheral nerves. In general, BoNT treatment intervenes pain in two ways: (i) direct way – by abolishing the contractile activity of the muscle (due to hyperactivity or sensitization of nociceptor by lowering the pH) and (ii) indirect way – by preventing the release of neurotransmitters other than acetylcholine (such as substance P (SP), calcitonin gene-related peptide (CGRP), somatostatin, serotonin, ATP, bradykinin (BKN) etc.) involved in sensitization and stimulation of muscle nociceptors, which lead to inhibition/enhancement of ascending/descending signals (such as chronic cases).

## Types of pain

Pain is generally evoked by potential noxious stimuli such as heat, chemical, or mechanical exposure. The pain during a disease is different from normal pain. Two types of nociceptive pain are usually distinguished based on point of origin: a pain emanating from the skin and deeper tissues (e.g. joints and muscles) is referred to as somatic pain, while pain emanating from the internal organs is referred to as visceral pain. Somatic pain is usually well localized, whereas visceral pain is harder to pinpoint.

Different types of pain have been classified according to their pathogenesis. The simple but very important distinction is acute and chronic pain. An acute pain is caused by injury to a specific part of the body, restricted to the injury site and is abolished after healing. Chronic pain is the condition when pain itself becomes a disease. Chronic pain persists for longer than 6 months and can arise even in the absence of any pathological trigger. A more scientific distinction would have three types of pain: (i) acute physiological nociceptive pain, which protects tissues from further damage eliciting withdrawal reflexes, (ii) pathophysiological nociceptive pain occurs when the tissue is inflamed or injured. It may appear as a spontaneous pain, hyperalgesia, or allodynia. (iii) Neuropathic pain results from injury or damage to neurons. Neuropathic pain often feels abnormal and may be combined with hyperalgesia and allodynia. Cause of neuropathic pains includes nerve or plexus damage, metabolic disease, or herpes zoster. Pain can be neuropathic or inflammatory, although neuropathy may involve inflammatory components and neuropathic components may contribute to inflammatory pain. Other types of pain include pain during surgery, cancer pain, pain during degenerative disease, or pain in the house of psychiatric disease.

## How nociceptors are activated and transmit signals?

Nociceptors respond to various stimuli which are generated by a variety of substances. These substances activate various channels (see below; Box 1). Globulins, protein kinases, arachidonic acid, histamine, nerve growth factor, SP, CGRP, potassium, serotonin, lactic acid, and acetylcholine are the substances generated or released in response to the stimulus. Nociceptors have ion channels for stimulus transduction, generate the action potential, and carry specific receptors. Let us discuss how these substances are involved in activation of nociceptors.

Box 1 **Schematic Diagram of General Pain Pathways**

Activation of receptor by thermal, mechanical, or chemical stimulation converts the received form of energy into chemical and electrical form, which is accessible to the brain. This process is known as transduction. Nociceptors are primary sensory neurons, which sense the stimuli capable of causing tissue injury. Transduction and processing of these stimuli at the conscious level produce the sensation of pain. Signal transduction happens at the free nerve ending, which is made of different fibers; A (large, broad, and myelinated) and C (small, thin, and non-myelinated). A-fibers are responsible for early, sharp, brief pain (type I; first pain), and C-fibers are responsible for dull, prolonged pain (type II, second pain). Nociceptors also include several ‘silent’ sensory neurons that only become responsive after a sensitizing stimulus – e.g. inflammation. A-fibers are divided into three; Aα, Aβ, and Aδ-fibers. Aα and Aβ are large diameter myelinated fibers, whereas Aδ-fibers are medium diameter, lightly myelinated fibers. Sensitivity of Aα and Aβ is high and is involved in light touch. Aδ-fibers are involved in mechanical, thermal, and chemical stimulation. C-fibers can be divided into two broad classes; (i) peptidergic: contains neuromodulators SP and CGRP, expresses trKA neurotrophin receptor (NGF responsive), terminate superficially in dorsal horn (I and II outer) and (ii) non-peptidergic lectin IB4, expresses purinoceptor 2X3 (p2X3) ATP receptor; receptor for GDNF family of neurotrophic factors, terminate in deeper substantia gelatinosa (II inner).

During injury, chemical substances are produced in the damaged area such as serotonin, BKN, histamine, and prostglandins. These substances stimulate the nociceptors giving the sensation of pain and they also are the cause of local edema or inflammation.

Neural impulses are carried along the peripheral nerve to the spinal cord. Majority of neurons project to the spinal cord through dorsal root (back side), but some may project through ventral root (front side; abdominal side). In the cord, there are three types of neurons; projection neurons, excitatory neurons, and inhibitory neurons. Projection neurons mainly relay to the higher brain center, excitatory neurons relay to the motor neurons that mediate spinal reflexes, and inhibitory neurons contribute to the regulation of nociceptive transmission. The myelinated afferents excite inhibitory neurons, and the non-myelinated nociceptors inhibit the inhibitory interneurons. The perceived pain is the net effect. There are several chemical substances in the cord related to the impulse transmission including SP, somatostatin, vasoactive intestinal peptide, glutamate, aspartate, and adenosine phosphate.

In structures with higher brain functions, such as thalamus and cerebral cortex, nociceptive signals are integrated with signals from a variety of psychological processes such as emotions, beliefs, expectations, perceived environmental demands, and memories of the past painful events. Higher brain structures are capable of modulating both nociceptor sensitivity and subsequent afferent transmission of sensory signals by the descending analgesic system, which finally send signals to motor neuron for further actions. In general, two kind of descending analgesic signals; opioid or endorphin mediated analgesic signals, and non-opioid analgesic signals.

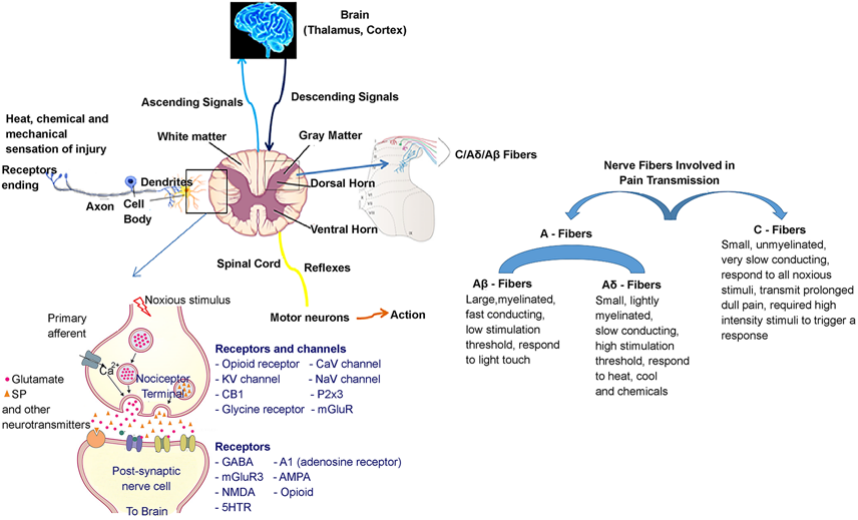

Primary afferent nerve fibers detect environmental stimuli (of a thermal, mechanical, or chemical nature). A set of molecules is involved in detecting stimuli and transduction into electrical signals. Human or animal demarcates between innocuous warmth and noxious heat. Several pieces of evidence suggest that transient receptor potential (TRP) ion channel family is involved in transducing heat [13]. TRP vanilloid receptor 1 (TRPV1), 1 of the 30 members of TRP family, is expressed in the majority of heat-sensitive nociceptors and acts as a molecular integrator of thermal and chemical stimuli [13–15]. However, the TRPV1 is not the sole ion channel responsible for heat transduction. Many other TRPV channel subtypes are also identified as candidates for heat transducers, such as TRPV 2, 3, and 4 [16,17]. Likewise, TRP menthol receptor 8 (TRPM8) ion channel was identified as a molecular integrator for the cold-related response. Interestingly, heat and cold detection mechanisms are distinctly organized into anatomical and functional entities [18]. As in the case of TRPV1 and heat, TRPM8 is also not solely involved in response to cold stimuli, other proteins such as TRPA1, voltage-gated sodium and potassium channels and two-pore potassium channel subfamily K (KCNK) potassium channels, co-ordinate with TRPM8 to fine-tune cold-related response [19–21].

A variety of mechanosensitive receptors can detect various mechanical stimuli and can be categorized according to their sensitivity. High-threshold mechanoreceptors include C-fibers and slowly adapting Aδ mechanoreceptor, and low-threshold mechanoreceptors include Aβ-fibers. In brief, Aβ-fibers are primarily associated with sensitivity to light touch, whereas C- and Aδ-fibers are primarily responsive to noxious mechanical stimulation.

Candidates for molecular integrators of mechanical stimulations are mec-4 and mec-10 (see Box 1) [22]. Other molecules are Mec-2 ortholog and stomatin-like protein 3 (SLP3) had been identified as low-threshold mechanical stimulation [23]. TRPV2 and TRPA1 were also identified as a detector of mechanical stimuli [24–27]. However, their physiological effects are unclear, and they do not appear as primary detectors of mechanical stimuli [28,29]. Overall, the molecular basis of mammalian mechanotransduction is far from clarified.

Chemonociception involves TRP channels as prominent molecular integrators. TRPV1, TRPM8, and TRPA1 were identified as receptors for capsaicin, menthol, and pungent ingredients in mustard/garlic/plants/isothiocyanates/thiosulphates, respectively [13,20,30–32]. However, how these reagents promote channel activity is still unknown. Endogenous chemical irritants, in response to physiological stress/tissue damage/metabolic activity, can act alone or in combination to sensitize nociceptors.

Once the primary afferent terminal transduces signals, these activate a variety of voltage-gated K^+^/Na^+^ channels (see Box 1). These channels generate action potentials that convey nociceptor signals from the synapse to the dorsal horn of the spinal cord. Voltage-gated calcium channels, which play a key role in neurotransmitter release, are important to generate pain or neurogenic inflammation. These channels are well-known targets of anesthetic drugs (Box 2). Conduction of signal starts from membrane depolarization, followed by activation of voltage-gated calcium channels, and is referred to as generator potential. Once generator potential reaches the threshold, it generates an action potential. This depolarization in nociceptors closes K^+^ channels and opens Na^+^ channels. There are several channels known to facilitate conduction and modulation of action potential. Three classes of ion channels are essential for rapid transduction:

- Channels that influence passive membrane properties (resting membrane resistance, membrane capacitance, and intracellular axial resistance),
- Channels that influence passive/active membrane properties (voltage-gated sodium channel),
- Channels that influence the repolarization.

Box 2 **Types of pain, their cellular targets, and treatment options**

- **Pain targets**

**Table:** 

Pain	Targets
Inflammatory pain (skin, joint, and viscera)	TRPV1, purinoceptor 2X3 (P2X3), BKN (1 and 2 receptor), EP (prostaglandin receptor 1), Na_v_1.8 and Na_v_1.9, protein kinase C and A (PKC/A)
Neuropathic pain (peripheral nerve damage by cold, hot, acid, or mechanical stimuli)	TRPM8, TRPV1, acid-sensitive ion channel (ASIC), prostaglandin receptor 1 (EP), Na_v_1.8, Na_v_1.9, Na_v_1.3, Na_v_β3, Kv1.4, α2AR (α2 adrenoceptor), Cavα2δ1
Central sensititzation (neuropathic/inflammatory)	Gly (glycine receptor), neurokinin 1 (NK1), EP (prostaglandin receptor 1), metabotrophic glutamate receptor, N-methyl-d-aspartate, prostaglandin E2, 5-hydroxytryptamine, Cavα2δ1, noradrenaline transporter, α2AR
- **Treatment options for chronic pain**

1. **Physical:** It includes normal activities, aqua fitness, physiotherapy, stretching, conditioning, weight training, splinting/taping, massage, chiropractic etc. Effective in regaining range of motion and strength, and no need for surgical incision. But it is time-intensive (sometimes requires multiple visits per week) and costly.
2. **Psychologic:** It includes hypnosis, stress management, cognitive-behavioral therapy, psychotherapy, meditation techniques etc. It may be helpful in cases where medication alone has not worked, and requires relatively short periods of time. But it may not be suitable for people with more complex mental health needs or learning difficulties, and depends on individual willingness.
3. **Pharmaologic:** It includes over the counter medications, topical medications, NSAIDs/COXIBs, immune modulators, tricyclic/antiepiliptic drugs/opioids, local anesthetics, muscle relaxants, N-methyl-d-aspartate blockers, CGRP blockers etc. This treatment is generally effective in most cases, but carries risk of GI, cardiovascular, renal, hematological, dramatological, and neurological adverse effects.
4. **Interventional:** It includes steroids, hyaluronan injections, trigger point therapy, nerve blocks, epidurals, orthopedic, botox, neurotomy, neurectomy, implantable simulators, implantable pain pumps. Generally, rapid-acting treatment and good option to avoid surgery, but it is not a long-term solution and may have detrimental effects on soft tissue and cartilage.

A set of action potentials that encode the intensity of a noxious stimulus applied within their receptive fields. There are 9 known genes in mammals for Na_v_, 10 Ca_v_, and 40 K_v_ channel proteins, many of which have multiple splice variants with different functional characteristics. Thus, there is an array of channels to modulate conduction of varying types of action potentials. The generated action potential can depend on various factors; threshold for action potential generation, duration and amplitude of the action potential, and maximal firing frequency. These factors are modulated by different voltage gated-ion channels, which is why these channels are therapeutic targets for pharmacological agents (see Box 2).

The cell body of primary afferent neurons is located in dorsal root ganglion (DRG), near to the dorsal horn of the spinal cord. The dorsal horn is made of various stacked laminae. Primary afferent axons may form a direct or indirect connection with three functional populations of dorsal horn neurons: (i) interneurons, (ii) propriospinal neurons, which extend over multiple spinal segments, and (iii) projection neurons, which participate in rostral transmission by extending axons beyond the spinal cord. All three components are interactive and are essential for the processing of nociceptive information. C-fibers make synapses in laminae I and II, Aδ-fibers in laminae I, II, and V, and Aβ-fibers in laminae III, IV, and V. Deeper laminae (V–VII, X) are thought to be involved in the sensory pathways arriving from deeper tissues such as muscles and the viscera. The predominant neurotransmitter release at the synapses between primary afferents and projection neurons is glutamate and aspartate, although notably the C-fibers release several neuropeptides such as substance SP, neurotensin, vasoactive intestinal peptide, cholecystokinin, and CGRP. The efficiency of transmission of these synapses can be altered via descending pathways and by local interneurons in the spinal cord. These modulatory neurons release a number of mediators that are either inhibitory (e.g. opioid peptides and glycine) or excitatory (e.g. nitric oxide and cholecystokinin), to provide a mechanism for enhancing or reducing awareness of sensations.

## How CNS responds to pain signals?

In general, pain pathway consists of three neuron chains that transmit pain signals from the periphery to the cerebral cortex. (i) First-order neurons, which have a cell body in DRG and two axons. One axon extends to the peripheral part and the other to the dorsal horn of the spinal cord. (ii) In the dorsal horn, axons synapse with second-order neurons to ascend signals through the lateral spinothalamic tract to the thalamus, medulla, and brainstem. (iii) In the thalamus second-order neuron synapse with third-order neurons, which ascends signal to higher brain areas, cerebral cortex. Spinothalamic tract (in thalamus), spinoreticular, and spinomesencephalic tracts (in the brainstem) are involved in this signal conduction. The nociceptive neurons from the ventrobasal complex, mainly project toward the primary somatosensory cortex and this pathway constitutes the lateral pain system which plays an important role in the discrimination of stimuli [33,34]. The affective motivational aspect of the pain is mediated by the medial pain pathway, which includes the intralaminar thalamic nuclei [35] and posterior aspect of ventromedial thalamic nuclei [36] that project to somatosensory cortex and limbic structures [37]. The most important endogenous analgesic system is the mesencephalic periaqueductal gray (PAG) matter. Although PAG is known for descending modulation of nociceptive information, it also affects the ascending projection by providing an alternative pathway for nociceptive sensory activity to reach diencephalic structures. In ascending transmission, PAG is involved via rostral connections to thalamic, and limbic structures, and caudal afferents projected to the rostral ventromedial medulla.

Although ascending transmission of signal from sensory neurons to the super structure of the brain is important, descending inhibitory pathways involve in modulating all kinds of sensory inputs. The descending modulatory system has four components: (i) the cortical and thalamic structures, (ii) the PAG of the midbrain, (iii) the rostral medulla and pons of the brain stem, and (iv) the medullary and spinal cord dorsal horn [38]. PAG receives descending inputs from cortex, amygdala, and hypothalamus, and is modified by ascending projections from the medulla, reticular formation, and spinal cord [38]. Several distinct rostral ventromedial medulla is involved in the descending nociceptive inhibition arising from PAG. In the spinal cord, dorsal horn has the same importance for antinociception as it does in the integration of noxious stimulation. Since dorsal horn neurons involve complex interactions and single neurons in this region can be influenced by several neurotransmitters. Several nociceptor inhibitory neurotransmitters are identified in dorsal horns, such as γ-aminobutyric acid (GABA), glycine, serotonin, norepinephrine, and the opioid peptides (enkephalins, endorphins, and dynorphins) [38,39]. Neurons with the nucleus raphe magnus have been shown to project to the spinal cord and dorsal horns and directly or indirectly enhance or diminish pain nociception [40].

However, pain pathways are a dual system at each level and signals reaching the CNS are a combination of two distinctive components of pain; sensory discriminative component (first pain) and the emotional component (second pain). Both components are carried separately and contribute to individual pain. The spinothalamic pathway is related to the sensory discriminatory component, and the second component uses a pathway from the dorsal horn that expresses the neurokinin 1 (NK1) receptor (NK1r) and terminates within the parabrachial area and PAG. However, some of the areas of the brain are neither purely sensory nor purely motor but instead are modulatory. These modulatory system controls and direct complex behaviors such as motivation, hunger, thirst, emotion, memory, or sleep. One group of modulatory neurons in brainstem uses serotonin and noradrenaline to modulate forebrain functions. Another group is located in nucleus of Meynert involved in arousal or attention behavior. Cholinergic neurons in the basal nucleus are connected to all portions of neocortex and influence cognitive and perceptual processes.

Although central modulation of the nociceptive activity of BoNT is largely unknown, several research results indicate that neurotransmitter receptors have linked GABA and opioid transmission to the central antinociceptive action of the toxin. Both GABA and opioid transmission have a role in the attenuation of sensory input transmitted to the dorsal horn [41–43]. The functional block of GABA inhibition by BoNT/A and BoNT/B in spinal cord suggests the central modulation of nociceptive activity. Since cholinergic neurons in structures with higher brain functions (see above) are involved in modulatory circuits, the effect of BoNT on these neurons will reveal some fascinating details about the regulation and function of these modulatory neurons.

## Effect of botulinum toxin on sensory neurons

There are seven serotypes (A–G) of *Clostridium botulinum* known, although only A, B, and E are known for human botulism. Botulinum toxin is the most lethal substance known to mankind (lethal dose for the human is 1 × 10^−8^ g/kg; 100 billion times more toxic than cyanide). Higher potency of the botulinum toxin molecule is attributed to several factors:

- The molecule is synthesized as a complex, in which toxin molecule is surrounded by several non-toxic proteins. These non-toxic proteins prevent the toxin molecule from various uncongenial physiological environments, which may be one of the reasons of higher bioavailability of toxin molecule.
- Despite being a large molecule, botulinum toxin molecules pass through the epithelial barrier very efficiently using the endogenous process known as transcytosis.
- Targets specifically neuronal cells by using the dual receptor mechanism.
- There are unique characteristics of the longevity of BoNT inside the cell. BoNT/A can survive for up to 6 months and BoNT/E can survive for up to 1 month.
- BoNTs cleave specifically soluble NSF attachment receptors (SNARE) proteins at the synapse terminal and prevent fusion of synaptic vesicle to membrane.
- Structural and functional uniqueness of endopeptidase domain.
- Cleavage of a small portion of SNARE protein is required for complete blockage of synaptic transmission.
- BoNT activity is not only limited to inhibition of soluble N-ethylamide associated protein-25 kDa (SNAP-25) mediated acetylcholine release.

Toxin molecule has been shown to inhibit the release of serotonin, dopamine, noradrenaline, glutamate, GABA, enkephalin, glycine, SP, ATP, CGRP, somatostatin, and neuronal nitric oxide synthase [44].

BoNT action on peripheral nerve terminal results due to the inhibition of neurotransmitter release, and this function of BoNT has immense therapeutical interest. Binding of the molecule to neuronal terminal starts with binding at the plasma membrane to polyganglioside and then to high affinity binding to a protein receptor, which govern the endocytosis of the molecule [45]. A 50-kDa catalytic domain, light chain (LC), is translocated from the endocytic vehicle into the cytosol by a pH-dependent process mediated by N-terminal (translocation domain) of the heavy chain. In the cytosol catalytic domain, a metalloprotease, hydrolyzes SNARE proteins [46–48]. SNARE proteins, which play an important role in Ca^2+^-dependent exocytosis, comprising three proteins: SNAP-25, vehicle-associated membrane protein, and syntaxin.

BoNT has been shown to block the neurotransmitter release from excitatory neurons more efficiently compared with inhibitory neurons [49]. Although both types of neurons efficiently internalize the BoNT molecule, the low level of SNAP-25 at the inhibitory terminals [50] or cleaved SNARE protein may in fact be acting as the negative regulators of exocytosis in those neurons [49,51,52]. Grumelli et al. [53] and Verderio et al. [54] showed that the reducing calcium concentration increases the sensitivity of BoNT/A toxin to inhibitory neurons. SNAP-25 level is higher in excitatory neurons and is a negative regulator of calcium channels [55] making BoNT/A more sensitive to excitatory neurons. Contrary to the widely accepted concept, BoNT molecule enters not only cholinergic neurons, they influence many other neuronal types. For example, glutamate release from astrocytes is reduced by BoNT [49,56,57].

It is a well-established fact that botulinum toxin is very effective in treating various neuromuscular and autonomic disorders. Although central modulation of the nociceptive activity of BoNT is largely unknown, several research results indicate that neurotransmitter receptors have linked GABA and opioid transmission to the central antinociceptive action of the toxin. The functional block of GABA inhibition by BoNT/A and BoNT/B in spinal cord suggests the central modulation of nociceptive activity. Since cholinergic neurons in structures with higher brain functions (see above) are involved in modulatory circuits, the effect of BoNT on these neurons will reveal some fascinating details about the regulation and function of these modulatory neurons.

Very little is known about the intracellular trafficking of BoNT in neurons. Although because of its large size (150 kDa), it is unlikely for this molecule to pass through the blood–brain barrier, there can be two other possibilities for its transport to the CNS: systemic spread or axonal retrograde/anterograde transport. Antonucci et al. [60] showed the cleavage of SNAP-25 on facial motor nucleus after the peripheral administration suggesting a possibility of retrograde transport to brainstem. However, the concentration used in this experiment was very high compared with a therapeutic dose (~150-fold [58]). Lawrence et al. [59] suggested that BoNT/A and BoNT/E spread within cell bodies and distal neuronal processes may occur due to passive diffusion. However, recent experiments disregard the first possibility [58], and axonal transport is the most likely mechanism for the distribution and transport of toxin in various regions of axons (possibly to CNS too) [60–62]. Restani et al. [63] showed a significant amount of SNAP-25 cleavage by BoNT/A in tectum after delivery into the eyes of the rat. Matak et al. [64] successfully demonstrated cleavage of SNAP-25 at distal sites from low-peripheral dose. Colchicine is an axonal transport blocker. Incubation of neurons with colchicine prior to exposing with Botulinum Toxin A prevents SNAP-25 cleavage [64], indicates that trafficking of BoNT/A is through axonal transport. BoNT/B is also demonstrated to have similar effects with spinal activation [65]. Axonal transport of BoNT raises several questions: (i) the role of SNARE proteins in the cellular physiology, other than in membrane fusion of synaptic vesicles at the synaptic terminal, is not clearly known. Recently, a few experiments have demonstrated their involvement in autophagosome–lysosome fusion in the later stage of autophagy [66]. Also, they have a role in vesicle formation and lipid bilayer mixing [67]. Although the protein machinery used in viral membrane fusion is different from SNARE proteins, extensive structural, and functional analysis can unravel the mechanism of viral fusion. Thus, SNARE proteins can have multiple roles in cellular physiology. Detailed knowledge of SNARE function can unravel more pathways for axonal transport of BoNT. (ii) What are the other possible targets of BoNT proteins? Not only acetylcholine release is inhibited by BoNT/A, arachidonic release, which is associated with acetylcholine release is also inhibited by BoNT/A [68]. Ray et al. [68] demonstrated that phospholipase A2 and arachidonic acid (important for neuroexocytosis, independent of SNAP-25) could be an alternative target of BoNT/A. (iii) What is the clinical significance of the axonal transport? Although several evidence have been presented for retrograde transport of BoNT/A, a more in-depth study is needed to understand its cellular and molecular mechanism. Also, specificity of BoNT/A action in CNS, and their dose dependence need to be established. These studies will be needed to establish the clinical utility of BoNT in sensitization, drug delivery, and pathophysiological conditions related to CNS. (iv) Is axonal transport of BoNT connected to pain pathways? If yes, then how? Several studies have indeed described changes at the level of the CNS in man as well as animals, treated intramuscularly with BoNT/A [69–71].

## Use of botulinum toxin as a pain medication

**Figure 1:**
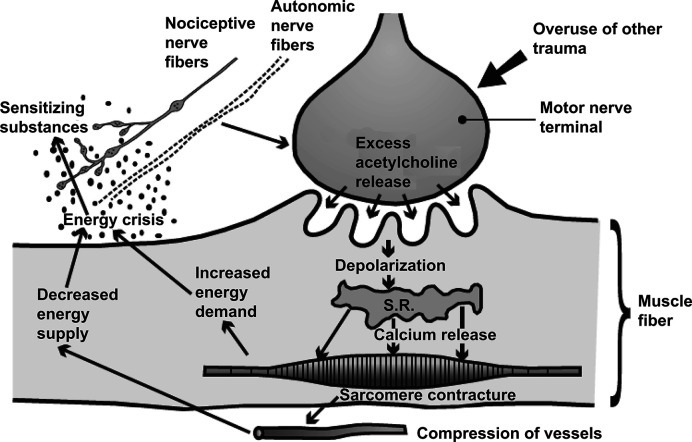
The endplate hypothesis of formation of MTrPs One of the reasons for the pain associated with a muscle tension or spasm is due to compression of the muscles for the pain. Formation of MTrP starts with a muscle lesion that leads to an excessive release of Ach into the synapse, which leads to depolarization. Depolarization is followed by a Ca^2+^ release from SR, which leads to the sliding of the myosin and actin filaments that form a local contracture. This local contracture compresses blood vessels that run along the muscle cells, which sensitizes nociceptors and generate pain. BoNT can be effective in hyperactive state of the muscle by abolishing the release of Ach in the neuromuscular junction that starts and maintain MTrPs (Mense [117]).Abbreviations: Ach, Acetylcholine.

Disorders in muscle tone could be a target for BoNT to be effective for pain. The muscle tone is determined by moving joints and involves the following two elements: the viscoelastic tone and the contractile activity. Viscoelastic tone is the specific muscle tone of resting muscle and is not dependent on the activity of the endplate. Such movements are not affected by BoNT. On the other hand, contractile activity is dependent on action potential generated by an electrogenic involuntary spasm (sudden involuntary muscle contraction) or contractions. The contractile activity includes voluntary contractions, and involuntary activity as in an incompletely relaxed muscle. The muscle contractions can be due to contraction knots of myofascial trigger points (MTrPs; Figure 1 ), spasm, and dystonia. Muscle nociceptors are activated by painful nociception, which activates homonymous α-motoneurons, leading to spasm in the muscle. These disorders of muscle contractions can be effectively treated with BoNT. BoNT can be effective in blocking the transmission between α-motoneuron and muscle cells. Botulinum toxin can also provide benefits related to effects on cholinergic control of nociceptive and antinociceptive systems. SNARE proteins, as a substrate of Botulinum Toxin, are also a regulator of various receptors at the plasma membrane, such as TRPV1, a protein involved in heat-induced nociceptive stimulation [72]. Interestingly, SNAP-25 is not exclusively localized in the presynaptic region, it is present in various parts of axons and dendrites [73]. In fact, all the components of SNARE complex may mediate various processes in the entire axonal compartment [73]. It is quite possible that SNARE proteins, present in another part of the neuron, may be affected by the botulinum toxin, affecting other cellular processes, such as apoptosis, neurotic sprouting, inhibition of acetylcholine mediated by arachidonic acid or lysophosphatidic acid pathways, and degradation of RhoB, which may not necessarily depend on the cleavage of SNARE [68,74–77].

Antonucci et al. [60], demonstrated that Botulinum Toxin A cleaves SNAP-25 distinct from the site of injection, suggesting the possibility of axonal transport of BoNT/A within central neurons and motoneurons. However, the dose (~450 pg/ml) used in this experiment is higher than the therapeutic dose (~3 pg/ml) [58]. Therefore, it can be hypothesized that the BoNT is effective in peripheral sensitization, which may lead to central sensitization, and the antinociceptive activity of botulinum toxin might be centrally mediated [78,79]. Although retrograde transport of botulinum toxin is still debatable, experiments performed by Back-Rojecky et al. [80] demonstrated that axonal transport is a prerequisite for the toxin nociceptive action. In their work, they could demonstrate the bilateral antinociceptive (both ipsilateral and contralateral) effect of the unilateral Botulinum Toxin A injection (peripheral). The bilateral antinociceptive action was confirmed by injecting colchicine, an axonal transport blocker. Administration of colchicine completely prevented the antinociceptive action of botulinum toxin. Obviously, the mechanism of the botulinum toxin antinociceptive action due to injection on the side of pain induction is not the same compared with injection in the contralateral side. Luvisetto et al. [5] showed the antinociceptive action of BoNT/A due to intrathecal (IT) injection, which was later confirmed by Back-Rojecky et al. [80].

Various studies have suggested that the BoNT could act selectively on extracranial afferents, trigeminal nociceptors, orofacial nociceptors, and other peripheral neurons. The involvement of SNAP-25 is important for the release of neurotransmitters and neuropeptides, many of which are involved in nociceptive processes, such as the CGRP. In animal models, peripheral injection of BoNT/A demonstrated significant antinociceptive sensitivity to mechanical and thermal stimuli. Aoki [81] demonstrated the inhibition of formalin-induced inflammatory pain by BoNT/A in rat model [3]. This antinociceptive activity was associated with the inhibition of formalin-induced release of glutamate, reflecting inhibition of release from primary afferent terminals. Altered expression of receptors and channels on the surface modifies the pain response, which can be blocked by administration of BoNT/A and ultimately attenuate the pain sensation.

Intradermal injection of BoNT/A reduces CGRP [82]. Based on results from several *in vitro* experiments, induction of nociceptive action of botulinum toxin might be due to the blockage or reduction in expression of neuropeptide transmitters, like SP, CGRP from the primary sensory neurons [44,83,84]. Botulinum toxin has been used ‘off-label’ in several chronic pain conditions. It has been observed that BoNT/A reduces pain in some conditions with not only concomitant muscle contraction, like in the painful dystonias but also in pain states not associated with muscle hypercontraction such as migraine [85], trigeminal neuralgia [86], neuropathic pain [12], refractory joint pain [87], and low-back pain [88].

Chronic musculoskeletal pain is a common cause of chronic pain. Non-randomized studies in patients with refractory joint pain after BoNT/A injection showed that the relief of pain after a few weeks [87,89], BoNT/A is also shown to provide relief to patients with chronic joint pain due to arthritis [84]. Ranoux et al. [12] observed analgesic effect of BoNT/A in patients with focal chronic neuropathic pain associated with allodynia, which may be due to the local peripheral effect on nerve fibers with possibility of central effects [90].

Conceptually, migraine pain is mediated through peripheral and central sensitization. Onabotulinumtoxin A is the only approved treatment for chronic migraine (CM) prevention [91,92]. It carries out this function in a two-phase action: in the first phase, it inhibits SNARE function and neurotransmitter release, and in the second phase, it has a neuromodulatory effect on receptors and ion channels. Although it was concluded that BoNT had no effect on episodic migraine, which may be indicating that neuromodulatory effects of BoNT takes some time to trigger effects on receptors and ion channels. Stimulation of trigeminal neurons releases CGRP and SP, which are associated with the pain in migraine [93]. TRPA1 and TRV1 are associated with CGRP-dependent pathways, and toxin effects on these channels, as well as inhibition of CGRP and SP release, could be another explanation of therapeutic efficacy of toxin in migraine.

Likewise, in arthritis, joint pain is reduced by 50% in 2/3 patients, who received BoNT/A injection [94]. Trigeminal neuralgia is several chronic pain syndromes and there are two ways available for its treatment: pharmacotherapy and neurosurgical procedures. A patient who does not respond well to pharmacotherapy, surgery is the only option. Subcutaneous injection of BoNT has provided complete relief in patients [86,95].

Interestingly, BoNT does not affect the normal pain threshold and is believed to affect only chronic or hypersensitive pain, and not the acute pain [3,96]. The lack of effect on acute nociception indicates and substantiates the arguments that the BoNTs effect on nociception is more than a simple blockage of the afferent terminal release. As in other medical treatments involving botulinum toxin, pain treatment has only few and tolerable side effects. Nevertheless, botulinum treatment invokes antigen response which hinders long-term use as a medicine [97,98]. Apart from antigen response, BoNT administration also significantly increases inflammatory cytokine level [99,100].

## Possible mechanisms of botulinum toxin as a pain medication

**Figure 2:**
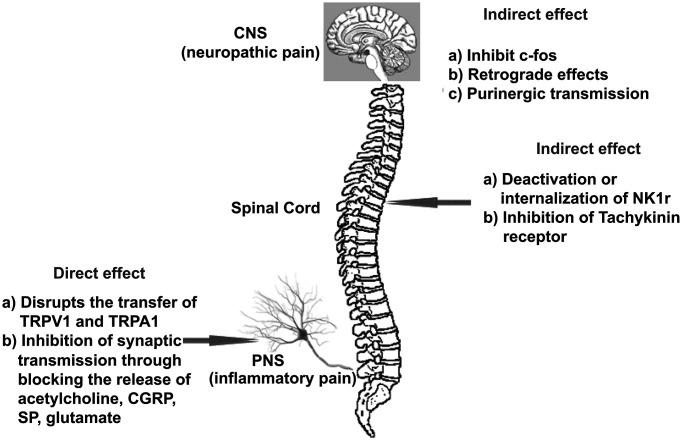
Possible effects of BoNT on PNS and CNS Effect of BoNT on PNS could be termed as direct effect on sensory neurons (nociceptors) either by disruption of expression of various receptors on neuronal membrane in presynaptic region or by inhibition of release of various neurotransmitters. The effect on CNS, on the other hand, is primarily due to the indirect effect either by deactivation/inhibition of signaling molecule or effect on purigenic transmission (due to ATP or nucleotides).

Botulinum toxin offers a viable option in various types of pain management compared with other existing treatments. Its usefulness is a welcome condition in this field because it is generally free from side effects except for the antigenic response. The BoNT can affect PNS directly and CNS indirectly by affecting various processes (Figure 2).

There is no definitive mechanism known how BoNT can modulate pain. But cumulative observation suggests a possible mechanism as follows:

### By blocking the release of neurotransmitters

**Figure 3:**
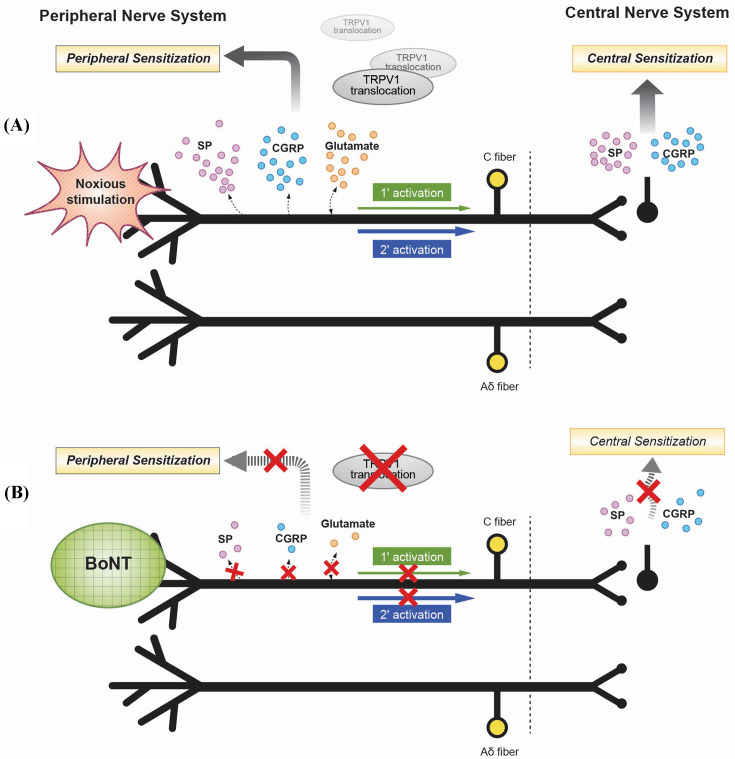
Schematics of peripheral and central sensitization and possible mechanisms of antinociceptive activity of BoNT. (**A**) Schematics of peripheral and central sensitization. Normal sensitization process occurs with the help of several neurotransmitters (SP, CGRP, and glutamate), ion channels, and receptors. Peripheral sensitization leads to central sensitization. (**B**) Possible mechanisms of antinociceptive activity of BoNT. BoNT not only inhibits release of peripheral and central neurotransmitters, but also affects the pain receptors (taken from Oh and Chung [131]).

Botulinum toxin may play a role in modulating pain pathways by impairing release of SP, glutamate, and CGRP (Figure 3). SNARE mobilization at the plasma membranes, leading to the terminal release of various afferent transmitters, such as acetylcholine, SP, CGRP. The release of SP and CGRP leads to protein extravasation [101,102]. Intraplantry administration of BoNT/A and BoNT/B resulted in an early plasma extravasation, suggesting a local block of the afferent peptide release [66,103–105]. Based on this concept, a hypothesis could be put forward as follows. SP is expressed in sensory nerve fibers and in the dorsal horn. It activates a receptor, NK1r, on the dorsal horn neurons. Activated NK1r is then internalized to dorsal horn, which correlates with SP release. Internalization of NK1r is evoked by afferent input and direct activation with IT SP. Internalization of NK1r is prevented by IT opiates and calcium channel blockers [106]. Intraplantry administration of botulinum toxin can prevent NK1r internalization by reducing SP release in the dorsal horn. BoNT did not affect the internalization of SP in case of IT administration. The NK1r reduces the behavioral response to pain and the pain-induced c-Fos activation in distinct brain areas which are intimately linked with nociceptive neurotransmission and the initiation and integration of central stress responses [107]. Mustafa et al. [108] have demonstrated that SP-BoNT/A LC conjugate can internalize BoNT/A LC selectively to NK1r-expressing neurons and delivers the LC inside the NK1r neurons. Internalized LC cleaves the SNAP-25 and can alleviate nociceptive behavior in a chemotherapy induced neuropathic pain [108]. Chaddock et al. [109–112] demonstrated several applications using conjugated proteins.

### By disrupting the transfer of pain receptors

**Figure 4:**
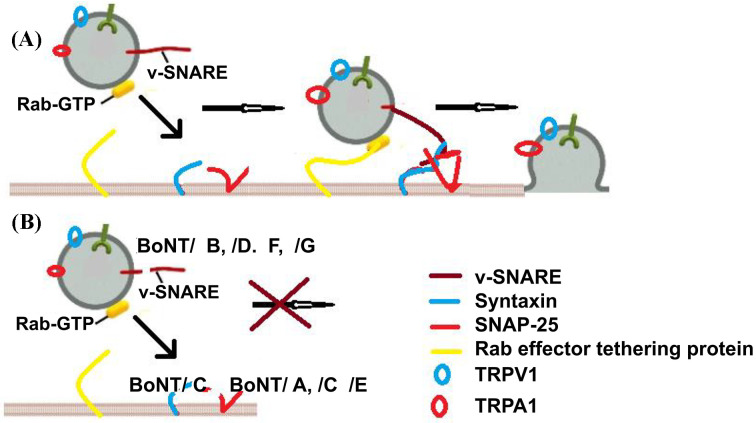
A possible mechanism for BoNT effectiveness on pain (**A**) Process showing the transfer of sensory receptor from synaptic vesicle through regular fusion process. Exocytosis process at the synapse is not only involved in neurotransmitter release, but also populates presynaptic regions with several pain receptors. (**B**) Impairment of fusion process by BoNT toxin which ultimately stops the transfer of receptors to the membrane. Inhibition of fusion vesicles reduces number of pain receptors at the synapse.

As mentioned above, there are two components that may play a role in botulinum toxin efficacy in pain modulation: impair of neurotransmitter release from peripheral sensory nerve and neuromodulatory effect on receptors and ion channels. The administration of botulinum toxin impairs the fusion of synaptic vesicles with the plasma membrane, which also carries various receptors, including receptors for pain. Peripheral administration of toxin disrupts the transfer of receptors, such as TRPV1 and TRPA1, to the synaptic membranes [113,114] (Figure 4). TRPV1 channels are present on various afferent neurons.

### By blocking the surface expression of receptors

BoNT/A blocks the surface expression of receptors and channels in primary afferent neurons. But it is not very clear if it affects the projection neurons and DRG. Peripheral injection of BoNT/A not only affects the neurotransmitter release in the presynaptic area, but it also affects the neurons beyond the synapses. Mika et al. [115] provided an evidence that the administration of BoNT/A relieves neuropathic pain. They also demonstrated that BoNT/A attenuates the up-regulation of NOS1, prodynorphin, and prepronociceptin mRNA levels in DRG, indicating BoNT/A role in chemical transduction and cellular signaling. After BoNT/A administration, they observed changes in expression of neuropeptides in the lumbar section of the spinal cord. Kim et al. [116] demonstrated that transcytosed BoNT/A evoked antinociceptive activity after peripheral injection, strengthening the hypothesis of the effect of BoNT/A on a site distal from the point of administration [60,63,116].

### By indirect desensitization of nociceptors

Another mechanism of pain where BoNT can be effective is muscle hyperactivity. Muscle hyperactivity starts with the formation of MTrP with a muscle lesion that leads to an excessive release of acetylcholine at the neuromuscular junction. Excessive acetylcholine causes a subthreshold depolarization of the postsynaptic muscle membrane which is followed by Ca^2+^ inflow from the sarcoplasmic reticulum (SR). Increased Ca^2+^ leads to muscle contraction due to sliding of myosin and actin filaments. Sliding of myosin and actin filaments compresses blood vessels. The combination of local ischemia and contraction causes a lack of oxygen, which releases BKN and other agents that sensitize nociceptors (Figure 1). By inhibiting acetylcholine release at the presynapse with BoNT helps in desensitization of nociceptors [117].

### By inducing indirect effect on pain processing pathways of CNS

Direct effect on the CNS from administration of BoNT is not evident, but it is possible indirectly through retrograde transfer (as described above). Moreover, peripheral administration of BoNT/A in reducing purinergic transmission is evident from clinical and laboratory research [118–120]. Activation of nerves influences early gene expression of c-fos in the postsynaptic terminal of neurons [121–123]. Studies have shown that BoNT (A and B) reduces c-fos expression upon inhibition of afferent fibers [65,124,125]. Immunodetection experiments also demonstrated the cleaved SNAP-25 on the spinal cord, suggesting the retrograde transport of BoNT/A after peripheral administration. Also, cleaved SNAP-25 has been found along all the neural nociceptive pathways [126] in BoNT-treated cells. This strongly suggests the possibility of combinatorial action at peripheral and central level by BoNT/A, which can modulate the pathological pain conditions.

### By affecting the signaling pathways of non-neuronal/supporting cells

Glial cells have critical contributions to the formation of synapses, neuronal plasticity, and protection against neurodegeneration. Growing evidence suggests that the neuronal connection has more than one cellular players. In chronic pain, if glial function can be regulated, then they can modulate the nociceptive signals. The main type of glial cells in the peripheral ganglia is satellite glial cells (SGCs). SNAP-25 and SNAP-23 are present in the SGCs and cleavage of these proteins by BoNT/A in SGCs inhibits the glutamate release [127]. Interaction of BoNT/A with non-neuronal cells suggest a potential analgesic mechanism of BoNT/A at the level of sensory ganglia. BoNT/A also inhibits the intracellular signaling pathways and the release of proinflammatory factors [128]. Although mechanism is not completely clear, but research indicated that BoNT/A exerts its anti-inflammatory action by inhibiting NF-kB, P38, and ERK1/2 activation in microglial cells and direct interaction with TLR2.

## Conclusion

The safety profile of BoNT is exemplary with minimal or no side effect [129,130]. Antibody formation and possible immune-related complications could be possible as a result of diffusion in the circulatory system or systemic spread. However, to develop antibodies higher amount of BoNT is required [131]. Furthermore, BoNT is being delivered through injections, including subcutaneous, intramuscular, or intra-articular. Comparison of different routes of injection might optimize the use of BoNT for the treatment of pain (the most appropriate dose of BoNT is unknown). With increasing use of BoNT as a therapeutic molecule, a new delivery system is urgently required. Oral or topical administration holds a better possibility for next generation of BoNT therapeutics.

In summary, BoNT/A can be an effective therapy in several types of pain management in which other treatments may be ineffective. It can also be effective in acute pain (with limited use). For example, pre-operative injection with BoNT/A has been shown to reduce and effectively manage post-operative pain [132]. The widely demonstrated antinociceptive activity of BoNT/A is primarily mediated by inhibition of neurotransmitter and neuropeptide release, and the inhibition of fusion mechanism in neuronal cells. However, detection of cleaved SNAP-25 at the site distal from the site of administration suggests the possibility of combinatorial action. BoNT fusion proteins with neuropeptides, such as SP-LC, hold promise for potential new innovative therapeutics for pain management. Also, BoNT effect on the non-neuronal cells holds the potential to explain the possible mechanism of BoNT utility in alleviating pain symptoms.

## Abbreviations

BKN: bradykinin
BoNT: botulinum neurotoxin
CGRP: calcitonin gene-related peptide
CNS: central nervous system
COXIB: cox2 inhibitor
DRG: dorsal root ganglion
ERK1: extracellular signal regulated kinases
GABA: γ-aminobutyric acid
GDNF: glial cell-derived neurotropic factor
GI: gastro intestinal
IB4: isolectine B4
IT: intrathecal
LC: light chain
MTrP: myofascial trigger point
NF-kB: nuclear factor kappa light chain enhancer of activated B cells
NGF: nerve growth factor
NK1r: neurokinin 1 receptor
NOS1: niric oxide synthase 1
NSAID: nonsteroidal anti-inflammatory drug
NSF: N-ethymaleimide sensitive fusion
PAG: periaqueductal gray matter
PNS: peripheral nervous system
SGC: satellite glial cell
SNAP-25: soluble N-ethylamide associated protein-25 kDa
SNARE: soluble NSF attachment receptor
SP: substance P
TLR2: toll-like receptor 2
TRP: transient receptor potential
TRPM8: TRP menthol receptor 8
TRPV1: TRP vanilloid receptor 1
trKA: tropomyosine receptor kinase A
